# Temperature measurements with two different IR sensors in a continuous-flow microwave heated system

**DOI:** 10.3762/bjoc.9.244

**Published:** 2013-10-10

**Authors:** Jonas Rydfjord, Fredrik Svensson, Magnus Fagrell, Jonas Sävmarker, Måns Thulin, Mats Larhed

**Affiliations:** 1 Department of Medicinal Chemistry, Uppsala University, Box 574, 751 23 Uppsala, Sweden; 2 Wavecraft AB, Bergsbrunnagatan 11, 753 23, Uppsala, Sweden; 3 Department of Mathematics, Uppsala University, Box 480, 751 06 Uppsala, Sweden

**Keywords:** continuous-flow, flow chemistry, heating, microwave, organic synthesis, temperature

## Abstract

In a continuous-flow system equipped with a nonresonant microwave applicator we have investigated how to best assess the actual temperature of microwave heated organic solvents with different characteristics. This is non-trivial as the electromagnetic field will influence most traditional methods of temperature measurement. Thus, we used a microwave transparent fiber optic probe, capable of measuring the temperature inside the reactor, and investigated two different IR sensors as non-contact alternatives to the internal probe. IR sensor 1 measures the temperature on the outside of the reactor whilst IR sensor 2 is designed to measure the temperature of the fluid through the borosilicate glass that constitutes the reactor wall. We have also, in addition to the characterization of the before mentioned IR sensors, developed statistical models to correlate the IR sensor reading to a correct value of the inner temperature (as determined by the internal fiber optic probe), thereby providing a non-contact, indirect, temperature assessment of the heated solvent. The accuracy achieved with these models lie well within the range desired for most synthetic chemistry applications.

## Introduction

In organic synthesis, being able to accurately determine the reaction temperature is often of utmost importance [1–3]. When using conventional heating the reaction mixture is typically heated from the outside, via the walls of the vessel. An internal temperature probe such as a thermometer or a thermocouple can in that case determine the temperature of the reaction mixture. In the last few decades the use of microwaves as a mode of heating has become increasingly popular in organic and medicinal chemistry [4–6], mostly owing to the development of computer-controlled dedicated reactors [7–10] that allow safe and rapid heating to high temperatures and elevated pressures. Microwave radiation directly heats the reaction mixture through two mechanisms; dipolar polarization and ionic conduction [11–13]. Compared to conventional heating this reverses the situation; in conventional heating the walls of the reaction vessel will be hotter than the reaction mixture whilst when using microwave heating the reaction mixture will have a higher temperature than the walls [8]. Unfortunately, under microwave radiation the usual methods to directly measure the internal liquid temperature of a reaction mixture such as thermocouples or mercury thermometers are affected by the electromagnetic field and will thus not be possible to use in the radiated zone [14–16]. In contrast, microwave transparent fiber optic probes can be used for correct temperature measurements. In a recent tutorial review Kappe highlighted the difficulties of temperature monitoring under microwave heating [16].

**Figure 1:**
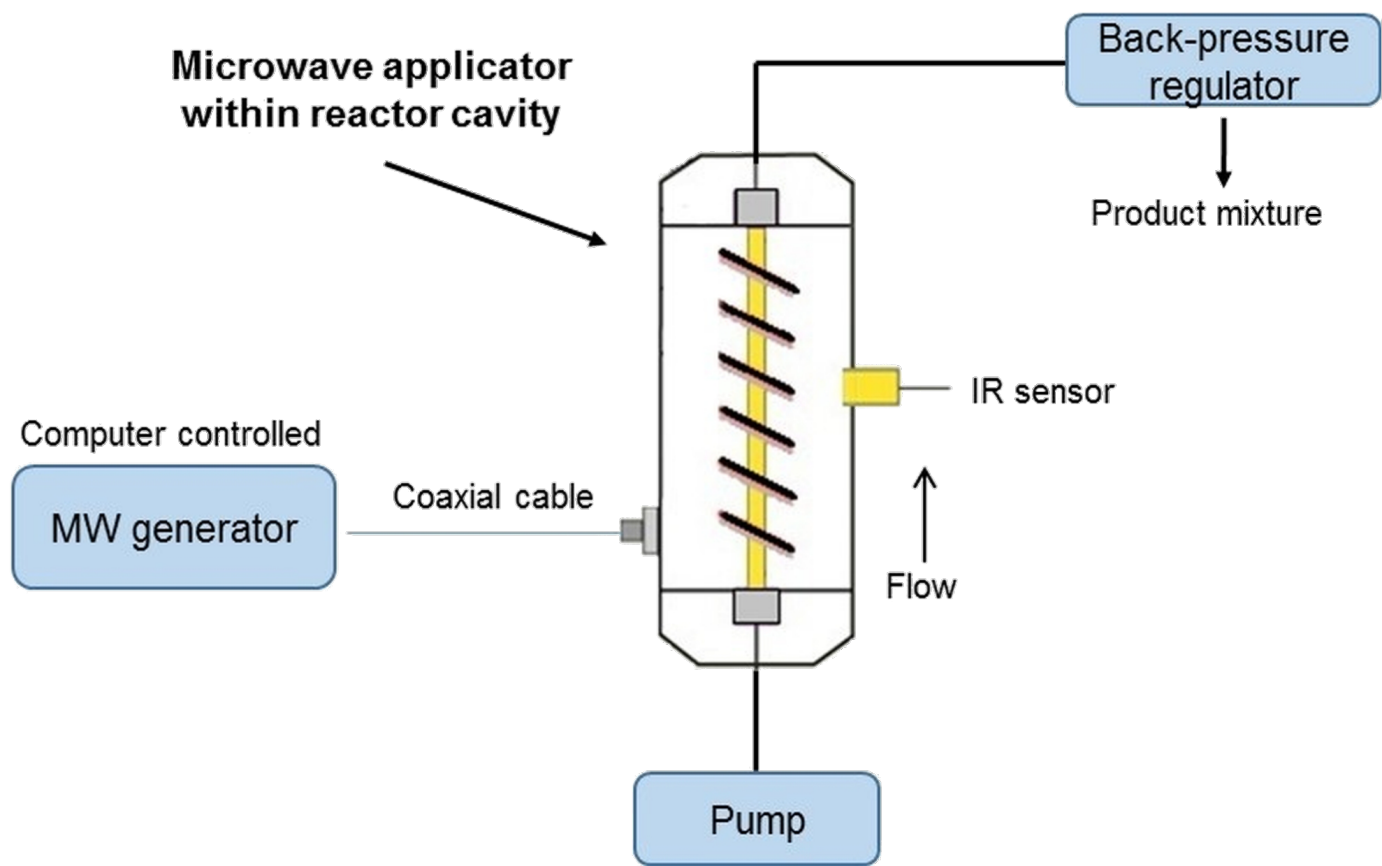
Instrument setup.

**Figure 2:**
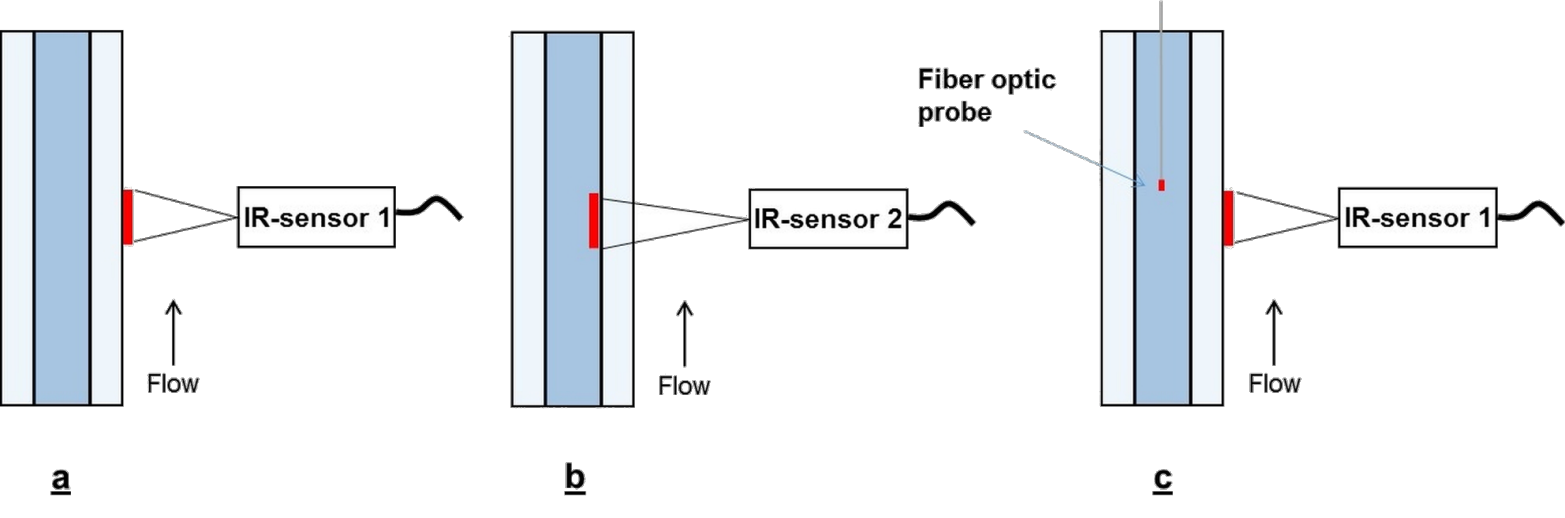
(a) Setup of system with temperature measurement by IR sensor 1. (b) Illustration of temperature measurement with IR sensor 2. (c) Simultaneous measurement of temperature by IR sensor 1 and an internal fiber optic probe.

We recently described the concept of a nonresonant microwave applicator for continuous-flow organic chemistry [17–18]. The current setup is depicted in Figure 1 and features a HPLC pump, a generator, a reactor cavity, an applicator and a Ø (ID) 3 mm × 200 mm borosilicate glass tube reactor. Nonresonant mode applicators has the advantage of avoiding hot and cold spots by creating a uniform axial field, which in this setup surrounds a linear tubular borosilicate glass reactor [17]. The temperature in this setup was measured using an external Optris CT infrared (IR) sensor with a LT22 sensing head (Optris GmbH, Berlin, Germany) situated in the reactor cavity which measure the temperature of the outer wall of the reactor (IR sensor 1, see Figure 2a). Acknowledging the problem of determining the temperature inside the reactor solely based on an IR sensor measuring the outer wall temperature, we decided to investigate the characteristics of this configuration with regards to what factors which affect the actual temperature of the flowing solvent in the reactor and, if possible, construct models to be able to relate the inside temperature to the outer temperature. Furthermore, we decided to include a second type of IR sensor with characteristics which could prove valuable in this and related applications (IR sensor 2, Optris CSmicro 3M, Optris GmbH, Berlin, Germany). IR sensor 2 has recently been introduced in the market and has a spectral response of 2.3 µm [19] which enable it to measure temperatures through borosilicate glass, effectively measuring on the actual fluid in the reactor (Figure 2b) [20].

## Results and Discussion

### Method

#### Measuring the internal temperature of the reactor

We opted for a fiber optic probe (Neoptix T1 Fiber Optic Temperature Probe, Neoptix Inc., Québec City) to be able to measure the temperature inside the glass reactor. The probe was positioned slightly above the measurement zone of the IR sensor (see Figure 2c, exemplified with IR sensor 1) to avoid possible problems with interference. IR sensor 1 or 2 was used to regulate the temperature and readings were then recorded using the fiber optic probe. This was done for five different temperatures (60, 80, 100, 120 and 140 °C) at four different flow rates (0.25, 0.5, 1, 2 mL/min) for nine different solvents categorized into groups depending on their tan δ value (High (tan δ > 0.5) [21]: isopropanol, methanol, DMSO; Medium (tan δ = 0.1–0.5) [21]: NMP, DMF, water; Low (tan δ < 0.1) [21]: Acetonitrile, THF, toluene). In total the full dataset with all measurements contains 173 data points for IR sensor 1 and 172 for IR sensor 2.

#### Data evaluation

The data collected were analyzed with regards to the influence of set temperature, flow rate and solvent to be able to distinguish between the two types of IR sensors and find the best way to correlate the “real” internal temperature of the reactor to the reading from the IR sensor in order to be able to apply a calibration to the IR sensor reading. We opted for linear multiple regression models using the full dataset, with all the measured data points. We also constructed several models with subsets of the data based on high, medium or low solvent absorption and one model where THF and toluene where excluded.

## Results

### Characterization of IR sensors

**Figure 3:**
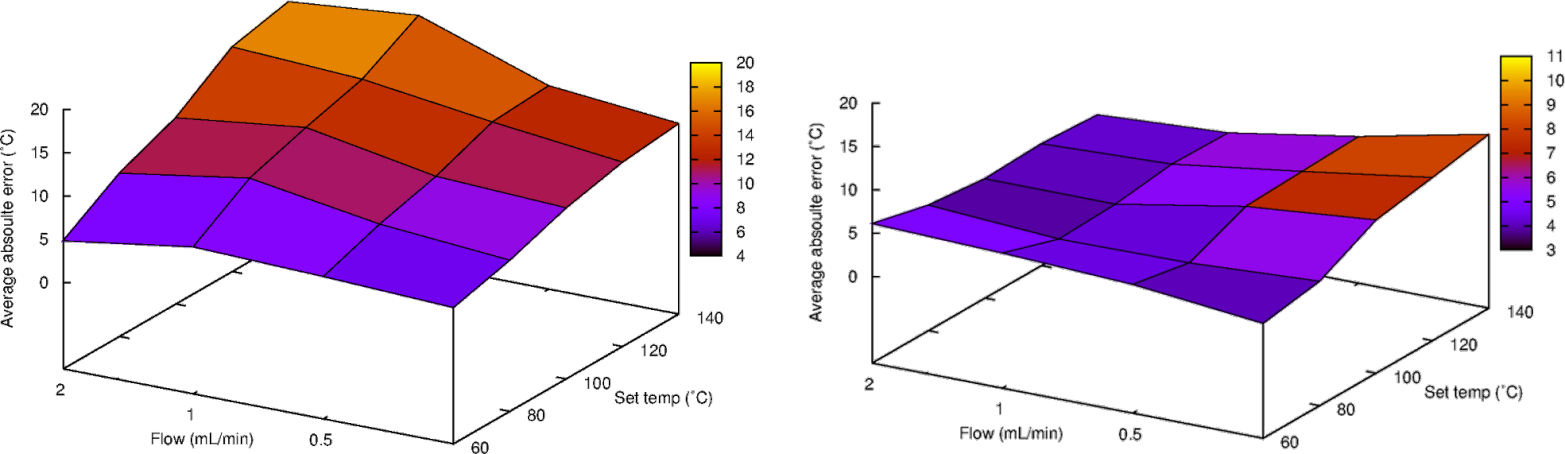
Average absolute errors in measurements for IR sensor 1 (left) and IR sensor 2 (right) plotted against flow and set temperature.

Examining the whole dataset for IR sensor 1 the average absolute error was 10.9 °C (Table 1). The average absolute errors were quite consistent for different flow rates, the extremes being 9.3 °C for 0.25 mL/min and 13.0 °C for 1.0 mL/min (Table 1). Examining the average absolute errors for different temperatures, an increase in average absolute error with temperatures were noted (also see Figure 3). The average absolute errors for the different solvents were also evaluated, which showed that THF and toluene have lower values (3.9 and 3.2 °C respectively) compared to the other solvents (all >10) (Table 1).

**Table 1:** Average absolute errors in measurements for the two IR sensors for all data and broken down by flow, set temperature and solvent.

Dataset	Average absolute error IR sensor 1 (°C)^a^	Average absolute error IR sensor 2 (°C)^a^

All data	10.9	5.4
Flow (mL/min)		

0.25	9.3	6.9
0.5	10.5	5.7
1	13.0	4.4
2	11.0	4.6
Set temperature (°C)		

60	6.0	5.0
80	9.1	4.0
100	11.3	5.4
120	13.7	6.2
140	15.2	6.6
Solvent		

isopropanol	11.0	4.2
methanol	12.9	5.7
DMSO	15.3	6.5
NMP	14.1	6.9
DMF	14.1	4.8
water	11.3	5.0
acetonitrile	10.5	4.4
THF	3.9	5.3
toluene	3.2	5.8

^a^Calculated as the average absolute difference between temperature measured by the IR sensor and the fiberoptic probe.

The average absolute error for the whole dataset for IR sensor 2 was lower than for IR sensor 1 (5.4 °C compared to 10.9 °C, Table 1). IR sensor 2 also gave substantially lower average absolute errors for most solvents, THF and toluene being the exceptions. The data indicate that higher flow rates produce a lower average absolute error for this sensor (Table 1 and Figure 3). However, no apparent correlation could be noticed for the average absolute error for different temperatures or solvents (Table 1).

### Model construction for IR sensor 1

A linear multiple regression model was constructed with the set temperature (corresponds to the IR sensor reading), flow rate and solvent properties (specific heat capacity, dipolar moment, dielectric constant and tan δ) as explanatory variables (Table 2, model 1). This full model provided an adjusted R^2^ value of 0.985 (Table 2, model 1). Removing non-significant variables (99.9% level) from this model gave a new model (Table 2, model 2) with an adjusted R^2^ value of 0.988.

**Table 2:** Linear multiple regression models for IR sensor 1.

Model	Adjusted R^2^	RSE^a^	Variable	Coefficient	p value

Sensor 1, model 1 All data	0.985	3.96	Intercept	−13.30169	6.41×10^−11^***
			set temperature	1.10228	< 2×10^−16^***
			flow rate	0.58598	0.2077
			tan δ	7.14174	6.69×10^−12^***
			dielectric constant	0.05027	0.0536
			dipolar moment	2.09342	3.23×10^−12^***
			specific heat capacity	1.45386	0.0364
Sensor 1, model 2 All data	0.988	3.999	Intercept	−14.51355	8.62×10^−16^***
			set temperature	1.1011	< 2×10^−16^***
			tan δ	7.42205	1.30×10^−12^***
			dipolar moment	2.40693	< 2×10^−16^***
			specific heat capacity	2.51959	1.14×10^−8^***
Sensor 1, model 3 Excluded THF and toluene	0.989	3.467	Intercept	−0.1051	0.923
			set temperature	1.12938	< 2×10^−16^***
Sensor 1, model 4 All data	0.965	5.91	Intercept	−1.47943	0.411
			set temperature	1.11195	< 2×10^−16^***
			flow	1.02674	0.138
Sensor 1, model 5 All data	0.965	5.932	Intercept	−0.40469	0.807
			set temperature	1.11043	< 2×10^−16^***
Sensor 1, model 6 High tan δ	0.986	3.774	Intercept	2.01917	0.264
			set temperature	1.11088	< 2×10^−16^***
Sensor 1, model 7 Medium tan δ	0.990	3.191	Intercept	−1.05619	0.493
			set temperature	1.14437	< 2×10^−16^***
Sensor 1, model 8 Low tan δ	0.959	6.076	Intercept	−0.6141	0.838
			set temperature	1.05544	< 2×10^−16^***
Sensor 1, model 9 High and medium tan δ	0.988	3.505	Intercept	0.51549	0.664
			set temperature	1.12717	< 2×10^−16^***

^a^Residual standard error. *** Significant at 99.9%level.

As it was noted in the characterization of IR sensor 1 there was a large difference in errors between weakly microwave absorbing THF and toluene compared to the rest of the dataset. Removing toluene and THF from the full dataset and developing a new model using all variables before taking away non-significant (99.9% level) variables resulted in a model with only the set temperature as a variable, providing an adjusted R^2^ value of 0.989 (Table 2, model 3).

Creating a less complicated model for the full dataset, disregarding solvent parameters and only taking into account set temperature and flow rate, shows that the flow rate is a non-significant variable (99.9% level), affording an adjusted R^2^ value of 0.965 (Table 2, model 4). Removing flow rate as a variable and constructing a new model gives an adjusted R^2^ value of 0.965 (Table 2, model 5). Creating models for high, medium and low absorbing solvents using only set temperature as a variable gives adjusted R^2^ values of 0.986, 0.990 and 0.959 respectively (Table 2, models 6–8). A model created for only high and medium absorbing solvents gives an adjusted R^2^ value of 0.988 (Table 2, model 9).

### Model construction for IR sensor 2

In accordance with the results for IR sensor 1, a linear multiple regression model was constructed with the set temperature, flow rate and solvent properties (specific heat capacity, dipolar moment, dielectric constant and tan δ) as explanatory variables (Table 3, model 1). This comprehensive model provided an adjusted R^2^ value of 0.975 with all variables being significant (99.9% level) (Table 3, model 1). A refined model with set temperature and flow rate as variables shows that the flow rate is a significant variable (99.9% level) and generates an adjusted R^2^ value of 0.963 (Table 3, model 2). Removing flow rate as a variable gives a model with an adjusted R^2^ value of 0.946 (Table 3, model 3). Creating models for high, medium and low absorbing solvents using only set temperature and flow rate as variables furnish adjusted R^2^ values of 0.983, 0.959 and 0.970, respectively (Table 3, models 4–6). A final model produced for only high and medium absorbing solvents gives an adjusted R^2^ value of 0.970 (Table 3, model 7).

**Table 3:** Linear multiple regression models for IR sensor 2.

Model	Adjusted R^2^	RSE^a^	Variable	Coefficient	P

Sensor 2, model 1 All data	0.975	4.127	Intercept	12.69142	1.93×10^−9^***
			set temperature	0.90804	< 2×10^−16^***
			flow rate	5.4351	< 2×10^−16^***
			tan δ	−6.92092	1.28×10^−10^***
			dielectric constant	0.12718	4.63×10^−6^***
			dipolar moment	−1.73721	1.38×10^−8^***
			specific heat capacity	−2.55026	5.17×10^−4^***
Sensor 2, model 2 All data	0.963	*4.987*	Intercept	4.5697	0.00317
			set temperature	0.9026	< 2×10^−16^***
			flow rate	5.1261	1.65×10^−15^***
Sensor 2, model 3 All data	0.946	6.002	Intercept	9.97626	1.62×10^−8^***
			set temperature	0.89482	< 2×10^−16^***
Sensor 2, model 4 High tan δ	0.983	*3.3*	Intercept	3.52478	0.0398
			set temperature	0.8785	< 2×10^−16^***
			flow	6.29357	5.42×10^−14^***
Sensor 2, model 5 Medium tan δ	0.959	*5.206*	Intercept	4.23114	0.135
			set temperature	0.89792	< 2×10^−16^***
			flow	5.70054	1.01×10^−6^***
Sensor 2, model 6 Low tan δ	0.970	*4.656*	Intercept	4.55598	0.085468
			set temperature	0.94315	< 2×10^−16^***
			flow	3.929	0.000389***
Sensor 2, model 7 High and medium tan δ	0.970	*4.428*	Intercept	3.88411	0.0189
			set temperature	0.88809	< 2×10^−16^***
			flow	5.98535	< 2×10^−16^***

^a^Residual standard error. *** Significant at 99.9%level.

## Discussion

Analyzing the collected data there seems to be a significant difference between the two IR sensors. Borosilicate-transparent IR sensor 2 has a lower mean absolute error before calibration using the linear model. Interestingly, although a linear model taking only the temperature as a variable gives similar residual standard errors for both sensors, adding flow as a variable provides a better model for IR sensor 2, while not being a significant variable for IR sensor 1. This supports the notion that IR sensor 2 is able to measure the temperature of the actual fluid stream in the reactor, as the flow rate should affect the readings more for this sensor than IR sensor 1 measuring the outer temperature of the reactor. Adding solvent properties provides better models for both sensors, suggesting that inherent properties of the solvents highly influence their characteristics in this setup, affecting factors such as their ability to transfer heat to the reactor walls and how well they are heated. In organic synthesis applications it is unlikely that the chemist knows the characteristics of the reaction mixture to such an extent to be able to input values of such specific solvent variables, therefore less complicated models using known variables are preferred. On that note, a large difference in average absolute error was discovered for low absorbing solvents such as toluene and THF when compared to the rest of the dataset for IR sensor 1. As it is unlikely that such low absorbing reaction mixtures (without polar components such as starting materials, reagents, catalysts or additives) will be of interest for the synthetic community, we decided to develop a model with only the set temperature as a variable to describe medium and high absorbing fluid systems (cutoff: tan δ > 0.1). For these systems, the new model was an improvement compared to the corresponding model for the full dataset, and this also constitutes our suggested model for assessing the internal temperature by external measurement (outer reactor surface) with IR sensor 1.

The same conclusions regarding the size of the dataset can be made for IR sensor 2. Excluding solvent parameters from the dataset is beneficial to make a model for fluid systems with tan δ > 0.1, however, for this sensor both set temperature and flow rate is included as variables.

IR sensor 1 calibrated with model 9 (Table 2) provides more accurate measurements for medium and high absorbing fluid systems than does IR sensor 2 calibrated with model 7: the former has lower residual standard error (3.505 °C compared to 4.428 °C; p = 0.0068 using an F-test), although the average absolute error is not significantly lower (2.826 °C compared to 3.446 °C; p = 0.0868 using a Mann–Whitney test). With these calibrations, the absolute errors range from 0.024 to 8.28 °C for IR sensor 1 and from 0.00 to 13.30 °C for IR sensor 2.

When also including low absorbing fluid systems in the comparison, the situation is reversed, with IR sensor 2 providing more accurate measurements after calibration. Comparing IR sensor 1 calibrated with model 5 and IR sensor 2 calibrated with model 2, the former has a larger residual standard error (5.932 °C compared to 4.987 °C; p = 0.0107 using an F-test), but there is no significant difference between the average absolute errors (4.392 °C compared to 3.918 °C; p = 0.3076 using a Mann–Whitney test). With these calibrations, the absolute errors range from 0.02 to 22.04 °C for IR sensor 1 and from 0.00 to 13.12 °C for IR sensor 2.

The practical aspects of the different methods of temperature measurements are also of great importance. An IR sensor does in that sense present a non-invasive measurement of temperature, be it external or internal. The fiber optic probe on the other hand will be invasive, and will disturb the flow in the reactor. Since it will also displace some of the reactor volume with its own body this might also to some extent affect the heating, as the power applied per volume unit will be higher where the fiber optic probe is located in the reactor. It is also quite sensitive to rough handling and may break, and, according to our experience, fiber optic probes are generally susceptible towards higher temperatures at elevated pressures in organic solvents.

Considering the above points the option of using the fiber optic probe as the standard tool for temperature measurements in continuous-flow synthesis appears less attractive. Although providing a direct measurement of the temperature in the reactor it is not robust enough for daily use and handling, especially under reaction conditions that may be relevant in organic synthesis applications [22–23].

**Figure 4:**
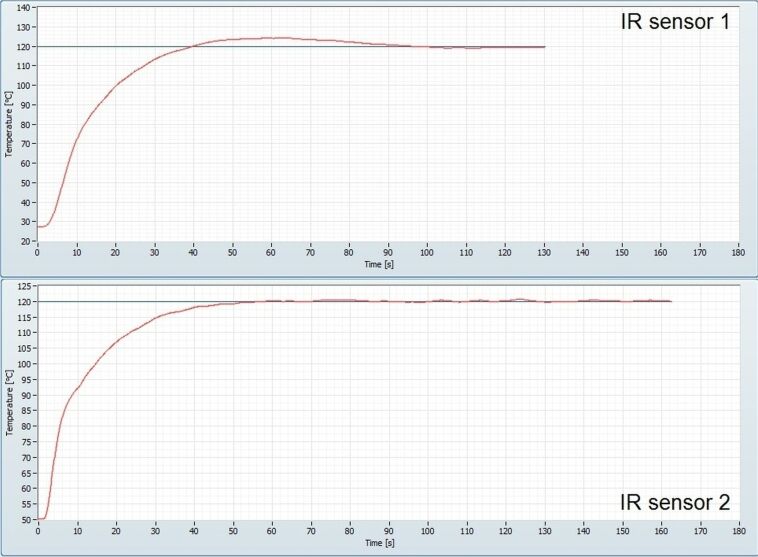
Heating profiles for IR sensor 1 (top) and IR sensor 2 (bottom) when heating isopropanol at a flow rate of 1 mL/min.

There is also one practical aspect that was noted as a difference between the two IR sensors, and that is that IR sensor 2, the one measuring directly on the fluid in the reactor, provides a much faster response to changes in temperature (Figure 4). This means practically, that when the software control adjusts power to reach set temperature it does not have to compensate for the lag that is created for IR sensor 1 when the reactor walls need to be heated. This characteristic might prove in handy for processes where a change in properties of the contents of the reactor happens suddenly as the control software can respond more directly by adjusting the power accordingly to maintain set temperature. This rapid response and the ability to measure the temperature through the borosilicate glass also, in the authors’ opinion, make an evaluation of IR sensor type 2 for use in batch instruments of prime importance.

**Figure 5:**
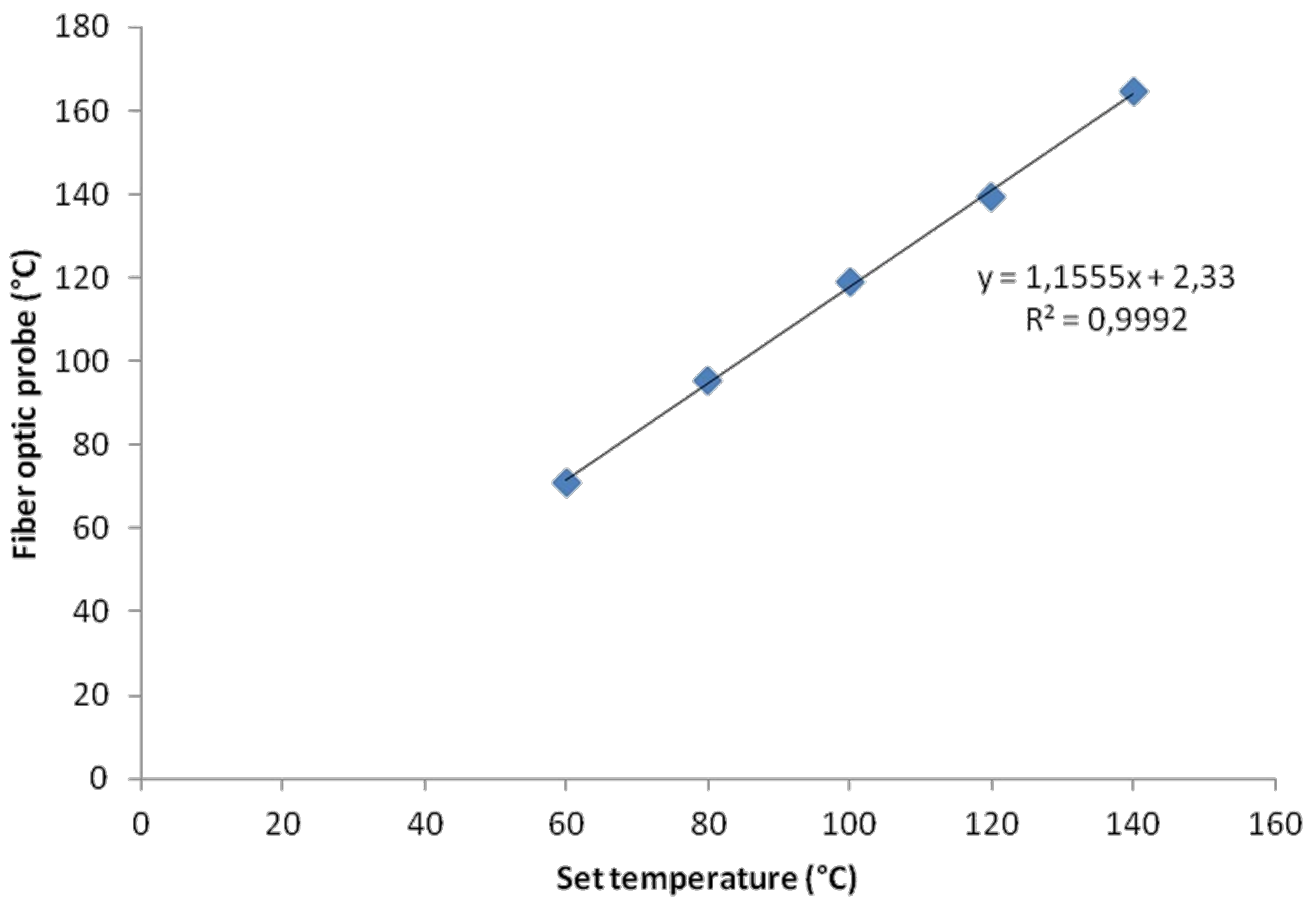
IR sensor 1, NMP, 1 mL/min.

For applications where such a setup as the one described is used for continuous production it is possible to, through a similar setup as the one we have used (Figure 1), simultaneously monitor the temperature with an IR sensor and a fiber optic probe. This way a temperature calibration based on the exact conditions can be performed, and the linearity within a specified set of conditions is in our experience very high (see for example, Figure 5). After the calibration has been performed the configuration can be changed again to only use an IR sensor for temperature measurement, applying the model found during the calibration to show the internal temperature. This is also our recommendation in general if knowledge of the exact temperature is of importance e.g for kinetic studies.

Finally, recommending one type of IR sensor is not trivial, as both have their advantages and disadvantages; IR sensor 1 provides somewhat better accuracy when applying models to assess the internal temperature while IR sensor 2 is closer to the internal temperature uncorrected. IR sensor 2 also provides faster response to changes in internal temperature which might be advantageous, whilst IR sensor 1 on the other hand is less direct since the changes in internal temperature must propagate through the reactor wall before being registered.

Using linear multiple regression models offer improvements over the uncalibrated readings, for which the average absolute errors were 10.9 °C for IR sensor 1 and 5.4 °C for IR sensor 2 (Table 1). Although not able to reduce the error down to the level of the IR sensors themselves [20,24] the accuracy achieved with these models lie well within the range desired for most medicinal and organic chemistry applications. Thus, our recommendation must be that the choice should be based on the characteristics of the IR sensor, rather than its performance, as both types of IR sensor tested perform good. Currently efforts in our lab focus on improving temperature calibration by on-the-fly determination of tan δ.

## Conclusion

It cannot be overstated that accurately measuring temperature under the influence of an electromagnetic field is not a trivial problem. In this evaluation we have used a fiber optic probe which allows the measurement of temperatures inside a reactor in a continuous-flow system using a nonresonant microwave heating device, and investigated two IR sensors as non-contact alternatives to this internal probe. Although differences were detected between the two IR sensors, in that IR sensor 2 provides a more direct measurement by being able to “see” through the borosilicate glass wall of the reactor, both sensors are, with a good degree of accuracy, able to assess the internal temperature after applying calibration by linear regression. Our study shows that temperature measurements using IR sensors in these systems can be improved using simple linear multiple regression models. In our case the models having the best balance between accuracy and simplicity used only one or two input variables. For IR sensor 1 the actual temperature was related only to the set temperature, and for IR sensor 2 the flow rate was also used as a variable. We also conclude that these models were improved when using only data for solvents with a tan δ > 0.1 (medium and high microwave absorbing solvents). In conclusion, we believe this investigation might be of interest for the future development and understanding of microwave heated continuous flow synthesis.

## Supporting Information

File 1 Experimental data.
