# Supplementary material for: Temperature measurements with two different IR sensors in a continuous-flow microwave heated system
Source: Beilstein J Org Chem. 2013 Oct 10;9:2079–87. doi: 10.3762/bjoc.9.244 (PMC3817514; doi:10.3762/bjoc.9.244)
Supplement: File 1 — Experimental data. [file Beilstein_J_Org_Chem-09-2079-s001.pdf]

Supporting Information

for

Temperature measurements with two different IR sensors in a continuous-flow microwave heated system

Jonas Rydfjord[1], Fredrik Svensson[1], Magnus Fagrell[2], Jonas Sävmarker[1], Måns Thulin[3] and Mats Larhed*[1]

Address: [1]Department of Medicinal Chemistry, Uppsala University, Box 574, 751 23 Uppsala, Sweden, [2]Wavecraft AB, Bergsbrunnagatan 11, 753 23, Uppsala, Sweden and [3]Department of Mathematics, Uppsala University, Box 480, 751 06 Uppsala, Sweden

Email: Mats Larhed - mats.larhed@orgfarm.uu.se

*Corresponding author

Experimental data

Chemicals

The solvents used were either reagent grade (methanol, DMSO, DMF and toluene) or HPLC grade (isopropanol, NMP, acetonitrile and THF) from commercial sources (Sigma-Aldrich, Fisher Scientific, Merck and LAB-SCAN) and free from stabilizers. They were used as provided without further purification or drying. The water used was purified using a Merck Millipore Synergy Ultrapure system to provide water of ultrapure (Type 1) quality.

Instrumentation

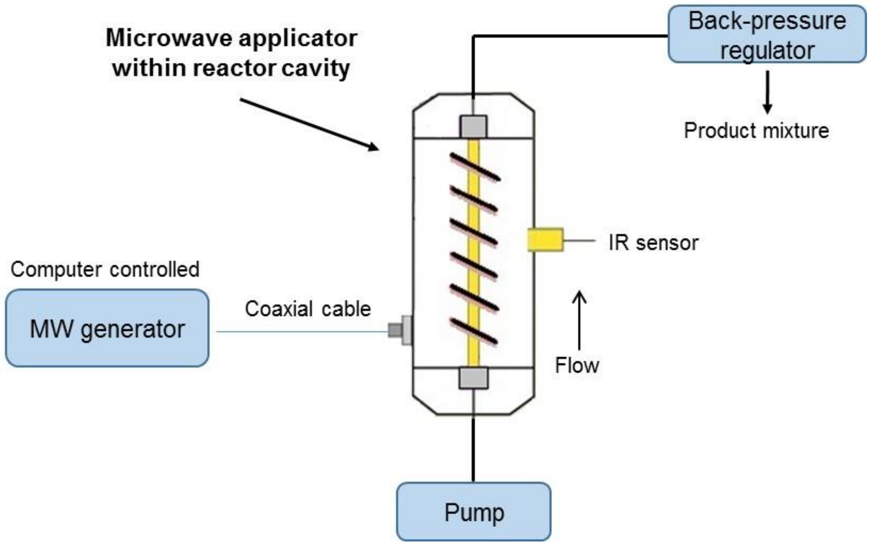

Schematic overview of the instrumentation

The MW generator, reactor, applicator and cavity are part of the ArrheniusOne™ system by WaveCraft AB. This system operates in a nonresonant mode and features a straight tube borosilicate glass reactor. The pump used was a LKB 2248 HPLC Pump. A U-609 back-pressure regulator assembly with a P-765 (500 psi) cartridge from Upchurch Scientific was used to regulate the pressure to 500 psi.

IR sensor 1 is a Optris CT sensor with a LT22 sensing head and IR sensor 2 is a Optris CSmicro 3M sensor. The fiber optic temperature probe used is a Neoptix T1 Fiber Optic Temperature Probe.

Data

All the measured data points for the two IR sensors. Tabulated data include Set temperature (Set, °C), Measured temperature (Measure, °C), Solvent, tan δ (Tan), specific heat capacity (SHC, $Jg^{-1}K^{-1}$), dielectric constant (DEC), dipole moment (DM, D).

Measured and tabulated data for IR sensor 1.

Set	Measure	Flow	Solvent Tan	SHC	DEC	DM	
60	63.5	0.25	Isopropanol	0.799	2.604	20.18	1.56
60	67.5	0.5	Isopropanol	0.799	2.604	20.18	1.56
60	67	1	Isopropanol	0.799	2.604	20.18	1.56
60	63.6	2	Isopropanol	0.799	2.604	20.18	1.56
80	88.9	0.25	Isopropanol	0.799	2.604	20.18	1.56
80	87.5	0.5	Isopropanol	0.799	2.604	20.18	1.56
80	91.7	1	Isopropanol	0.799	2.604	20.18	1.56
80	89.7	2	Isopropanol	0.799	2.604	20.18	1.56
100	114.6	0.25	Isopropanol	0.799	2.604	20.18	1.56
100	115.8	0.5	Isopropanol	0.799	2.604	20.18	1.56
100	114.9	1	Isopropanol	0.799	2.604	20.18	1.56

100	112.1	2	Isopropanol	0.799	2.604	20.18	1.56
120	129.5	0.25	Isopropanol	0.799	2.604	20.18	1.56
120	135.4	0.5	Isopropanol	0.799	2.604	20.18	1.56
120	134.2	1	Isopropanol	0.799	2.604	20.18	1.56
120	133.6	2	Isopropanol	0.799	2.604	20.18	1.56
140	152.5	0.25	Isopropanol	0.799	2.604	20.18	1.56
140	152.3	0.5	Isopropanol	0.799	2.604	20.18	1.56
140	154.5	1	Isopropanol	0.799	2.604	20.18	1.56
140	152	2	Isopropanol	0.799	2.604	20.18	1.56
60	68.2	0.25	Methanol	0.659	2.531	33	1.7
60	68.8	0.5	Methanol	0.659	2.531	33	1.7
60	69.7	1	Methanol	0.659	2.531	33	1.7
60	65.7	2	Methanol	0.659	2.531	33	1.7
80	93.7	0.25	Methanol	0.659	2.531	33	1.7
80	94.5	0.5	Methanol	0.659	2.531	33	1.7
80	94.8	1	Methanol	0.659	2.531	33	1.7
80	92.4	2	Methanol	0.659	2.531	33	1.7
100	120.1	0.25	Methanol	0.659	2.531	33	1.7
100	116.7	0.5	Methanol	0.659	2.531	33	1.7
100	114.9	1	Methanol	0.659	2.531	33	1.7
100	111.3	2	Methanol	0.659	2.531	33	1.7
120	136.5	0.25	Methanol	0.659	2.531	33	1.7
120	136	0.5	Methanol	0.659	2.531	33	1.7
120	135.6	1	Methanol	0.659	2.531	33	1.7
120	131.4	2	Methanol	0.659	2.531	33	1.7
140	151	0.25	Methanol	0.659	2.531	33	1.7
140	152	0.5	Methanol	0.659	2.531	33	1.7
140	153.5	1	Methanol	0.659	2.531	33	1.7
140	152	2	Methanol	0.659	2.531	33	1.7
60	68.2	0.25	DMSO	0.825	1.958	47.24	3.96
60	70.1	0.5	DMSO	0.825	1.958	47.24	3.96
60	67	1	DMSO	0.825	1.958	47.24	3.96
60	66.2	2	DMSO	0.825	1.958	47.24	3.96
80	88.9	0.25	DMSO	0.825	1.958	47.24	3.96
80	94.8	0.5	DMSO	0.825	1.958	47.24	3.96
80	92.9	1	DMSO	0.825	1.958	47.24	3.96
80	91	2	DMSO	0.825	1.958	47.24	3.96
100	114.4	0.25	DMSO	0.825	1.958	47.24	3.96
100	109	0.5	DMSO	0.825	1.958	47.24	3.96
100	118.3	1	DMSO	0.825	1.958	47.24	3.96
100	116.3	2	DMSO	0.825	1.958	47.24	3.96
120	134.5	0.25	DMSO	0.825	1.958	47.24	3.96
120	135.8	0.5	DMSO	0.825	1.958	47.24	3.96
120	142.6	1	DMSO	0.825	1.958	47.24	3.96
120	140.4	2	DMSO	0.825	1.958	47.24	3.96
140	163.8	0.25	DMSO	0.825	1.958	47.24	3.96
140	162	0.5	DMSO	0.825	1.958	47.24	3.96
140	166.6	1	DMSO	0.825	1.958	47.24	3.96
140	164	2	DMSO	0.825	1.958	47.24	3.96
60	65.4	0.25	NMP	0.275	3.105	32.55	4.1
60	65.8	0.5	NMP	0.275	3.105	32.55	4.1
60	71.1	1	NMP	0.275	3.105	32.55	4.1
60	67.3	2	NMP	0.275	3.105	32.55	4.1

80	87.2	0.25	NMP	0.275	3.105	32.55	4.1
80	87.2	0.5	NMP	0.275	3.105	32.55	4.1
80	95.2	1	NMP	0.275	3.105	32.55	4.1
80	92.2	2	NMP	0.275	3.105	32.55	4.1
100	110.1	0.25	NMP	0.275	3.105	32.55	4.1
100	112.1	0.5	NMP	0.275	3.105	32.55	4.1
100	119.1	1	NMP	0.275	3.105	32.55	4.1
100	116	2	NMP	0.275	3.105	32.55	4.1
120	137.7	0.25	NMP	0.275	3.105	32.55	4.1
120	136.9	0.5	NMP	0.275	3.105	32.55	4.1
120	139.5	1	NMP	0.275	3.105	32.55	4.1
120	139.5	2	NMP	0.275	3.105	32.55	4.1
140	152.5	0.25	NMP	0.275	3.105	32.55	4.1
140	160.3	0.5	NMP	0.275	3.105	32.55	4.1
140	164.5	1	NMP	0.275	3.105	32.55	4.1
140	161.6	2	NMP	0.275	3.105	32.55	4.1
60	62.8	0.25	DMF	0.161	2.06	38.25	3.82
60	66.7	0.5	DMF	0.161	2.06	38.25	3.82
60	70.3	1	DMF	0.161	2.06	38.25	3.82
60	67.9	2	DMF	0.161	2.06	38.25	3.82
80	89.7	0.25	DMF	0.161	2.06	38.25	3.82
80	95	0.5	DMF	0.161	2.06	38.25	3.82
80	95.5	1	DMF	0.161	2.06	38.25	3.82
80	93.9	2	DMF	0.161	2.06	38.25	3.82
100	111.3	0.25	DMF	0.161	2.06	38.25	3.82
100	116.1	0.5	DMF	0.161	2.06	38.25	3.82
100	116.3	1	DMF	0.161	2.06	38.25	3.82
100	113.8	2	DMF	0.161	2.06	38.25	3.82
120	133.6	0.25	DMF	0.161	2.06	38.25	3.82
120	139.5	0.5	DMF	0.161	2.06	38.25	3.82
120	140.1	1	DMF	0.161	2.06	38.25	3.82
120	136.5	2	DMF	0.161	2.06	38.25	3.82
140	152	0.25	DMF	0.161	2.06	38.25	3.82
140	158.7	0.5	DMF	0.161	2.06	38.25	3.82
140	163.1	1	DMF	0.161	2.06	38.25	3.82
140	159.2	2	DMF	0.161	2.06	38.25	3.82
60	68	0.25	Water	0.123	4.18	80.1	1.8546
60	69.1	0.5	Water	0.123	4.18	80.1	1.8546
60	65.5	1	Water	0.123	4.18	80.1	1.8546
60	62.2	2	Water	0.123	4.18	80.1	1.8546
80	86.4	0.25	Water	0.123	4.18	80.1	1.8546
80	90.8	0.5	Water	0.123	4.18	80.1	1.8546
80	91.7	1	Water	0.123	4.18	80.1	1.8546
80	90.4	2	Water	0.123	4.18	80.1	1.8546
100	108.5	0.25	Water	0.123	4.18	80.1	1.8546
100	113.9	0.5	Water	0.123	4.18	80.1	1.8546
100	113.3	1	Water	0.123	4.18	80.1	1.8546
100	109.8	2	Water	0.123	4.18	80.1	1.8546
120	132.4	0.25	Water	0.123	4.18	80.1	1.8546
120	135.8	0.5	Water	0.123	4.18	80.1	1.8546
120	136.8	1	Water	0.123	4.18	80.1	1.8546
140	154	0.25	Water	0.123	4.18	80.1	1.8546
140	156.8	0.5	Water	0.123	4.18	80.1	1.8546

140	158.8	1	Water	0.123	4.18	80.1	1.8546
60	65.3	0.25	Acetonitrile	0.062	2.229	36.64	3.92
60	64.8	0.5	Acetonitrile	0.062	2.229	36.64	3.92
60	65.2	1	Acetonitrile	0.062	2.229	36.64	3.92
60	65	2	Acetonitrile	0.062	2.229	36.64	3.92
80	87.5	0.25	Acetonitrile	0.062	2.229	36.64	3.92
80	85.4	0.5	Acetonitrile	0.062	2.229	36.64	3.92
80	88.2	1	Acetonitrile	0.062	2.229	36.64	3.92
80	89.2	2	Acetonitrile	0.062	2.229	36.64	3.92
100	108.2	0.25	Acetonitrile	0.062	2.229	36.64	3.92
100	106	0.5	Acetonitrile	0.062	2.229	36.64	3.92
100	112.5	1	Acetonitrile	0.062	2.229	36.64	3.92
100	112.5	2	Acetonitrile	0.062	2.229	36.64	3.92
120	130.3	0.25	Acetonitrile	0.062	2.229	36.64	3.92
120	131.5	0.5	Acetonitrile	0.062	2.229	36.64	3.92
120	136.8	1	Acetonitrile	0.062	2.229	36.64	3.92
120	134.6	2	Acetonitrile	0.062	2.229	36.64	3.92
140	155.6	0.25	Acetonitrile	0.062	2.229	36.64	3.92
140	155.9	0.5	Acetonitrile	0.062	2.229	36.64	3.92
140	158.9	1	Acetonitrile	0.062	2.229	36.64	3.92
140	156	2	Acetonitrile	0.062	2.229	36.64	3.92
60	64.8	0.25	THF	0.047	1.72	7.52	1.75
60	63.4	0.5	THF	0.047	1.72	7.52	1.75
60	64.9	1	THF	0.047	1.72	7.52	1.75
60	59.9	2	THF	0.047	1.72	7.52	1.75
80	84.5	0.25	THF	0.047	1.72	7.52	1.75
80	82	0.5	THF	0.047	1.72	7.52	1.75
80	87	1	THF	0.047	1.72	7.52	1.75
80	80.8	2	THF	0.047	1.72	7.52	1.75
100	99.3	0.25	THF	0.047	1.72	7.52	1.75
100	100.4	0.5	THF	0.047	1.72	7.52	1.75
100	108.7	1	THF	0.047	1.72	7.52	1.75
100	100.7	2	THF	0.047	1.72	7.52	1.75
120	115.6	0.25	THF	0.047	1.72	7.52	1.75
120	117.1	0.5	THF	0.047	1.72	7.52	1.75
120	129.2	1	THF	0.047	1.72	7.52	1.75
140	136.1	0.25	THF	0.047	1.72	7.52	1.75
140	141.5	0.5	THF	0.047	1.72	7.52	1.75
140	150.3	1	THF	0.047	1.72	7.52	1.75
60	65.7	0.25	Toluene	0.04	1.707	2.379	0.37
60	62.1	0.5	Toluene	0.04	1.707	2.379	0.37
60	62.4	1	Toluene	0.04	1.707	2.379	0.37
60	54.6	2	Toluene	0.04	1.707	2.379	0.37
80	79.3	0.25	Toluene	0.04	1.707	2.379	0.37
80	81.8	0.5	Toluene	0.04	1.707	2.379	0.37
80	83.8	1	Toluene	0.04	1.707	2.379	0.37
80	80.4	2	Toluene	0.04	1.707	2.379	0.37
100	99.7	0.25	Toluene	0.04	1.707	2.379	0.37
100	105	0.5	Toluene	0.04	1.707	2.379	0.37
100	101.8	1	Toluene	0.04	1.707	2.379	0.37
100	88.6	2	Toluene	0.04	1.707	2.379	0.37
120	117	0.25	Toluene	0.04	1.707	2.379	0.37
120	124	0.5	Toluene	0.04	1.707	2.379	0.37

120	121.5	1	Toluene	0.04	1.707	2.379	0.37
140	136.7	0.25	Toluene	0.04	1.707	2.379	0.37
140	142.3	0.5	Toluene	0.04	1.707	2.379	0.37

Measured and tabulated data for IR sensor 2.

Set	Measure	Flow	Solvent	Tan	SHC	DEC	DM
60	60.1	0.25	Isopropanol	0.799	2.604	20.18	1.56
60	64.6	0.5	Isopropanol	0.799	2.604	20.18	1.56
60	66.6	1	Isopropanol	0.799	2.604	20.18	1.56
60	67	2	Isopropanol	0.799	2.604	20.18	1.56
80	75.4	0.25	Isopropanol	0.799	2.604	20.18	1.56
80	78.4	0.5	Isopropanol	0.799	2.604	20.18	1.56
80	81	1	Isopropanol	0.799	2.604	20.18	1.56
80	84.4	2	Isopropanol	0.799	2.604	20.18	1.56
100	92.5	0.25	Isopropanol	0.799	2.604	20.18	1.56
100	93.5	0.5	Isopropanol	0.799	2.604	20.18	1.56
100	96.1	1	Isopropanol	0.799	2.604	20.18	1.56
100	101	2	Isopropanol	0.799	2.604	20.18	1.56
120	113	0.25	Isopropanol	0.799	2.604	20.18	1.56
120	113.1	0.5	Isopropanol	0.799	2.604	20.18	1.56
120	116.3	1	Isopropanol	0.799	2.604	20.18	1.56
120	120	2	Isopropanol	0.799	2.604	20.18	1.56
140	134.4	0.25	Isopropanol	0.799	2.604	20.18	1.56
140	134.3	0.5	Isopropanol	0.799	2.604	20.18	1.56
140	137	1	Isopropanol	0.799	2.604	20.18	1.56
140	142.6	2	Isopropanol	0.799	2.604	20.18	1.56
60	57.7	0.25	Methanol	0.659	2.531	33	1.7
60	60.3	0.5	Methanol	0.659	2.531	33	1.7
60	62.7	1	Methanol	0.659	2.531	33	1.7
60	64.7	2	Methanol	0.659	2.531	33	1.7
80	72.5	0.25	Methanol	0.659	2.531	33	1.7
80	74.7	0.5	Methanol	0.659	2.531	33	1.7
80	78.7	1	Methanol	0.659	2.531	33	1.7
80	81.6	2	Methanol	0.659	2.531	33	1.7
100	88.5	0.25	Methanol	0.659	2.531	33	1.7
100	89	0.5	Methanol	0.659	2.531	33	1.7
100	96.2	1	Methanol	0.659	2.531	33	1.7
100	99.4	2	Methanol	0.659	2.531	33	1.7
120	108.3	0.25	Methanol	0.659	2.531	33	1.7
120	108.5	0.5	Methanol	0.659	2.531	33	1.7
120	114.2	1	Methanol	0.659	2.531	33	1.7
120	118.7	2	Methanol	0.659	2.531	33	1.7
140	126.2	0.25	Methanol	0.659	2.531	33	1.7
140	128.5	0.5	Methanol	0.659	2.531	33	1.7
140	134.7	1	Methanol	0.659	2.531	33	1.7
140	139.6	2	Methanol	0.659	2.531	33	1.7
60	61.1	0.25	DMSO	0.825	1.958	47.24	3.96
60	64.3	0.5	DMSO	0.825	1.958	47.24	3.96
60	66.9	1	DMSO	0.825	1.958	47.24	3.96
60	67.8	2	DMSO	0.825	1.958	47.24	3.96
80	74.7	0.25	DMSO	0.825	1.958	47.24	3.96
80	79	0.5	DMSO	0.825	1.958	47.24	3.96
80	83.7	1	DMSO	0.825	1.958	47.24	3.96

80	86.3	2	DMSO	0.825	1.958	47.24	3.96
100	88.4	0.25	DMSO	0.825	1.958	47.24	3.96
100	94	0.5	DMSO	0.825	1.958	47.24	3.96
100	100.2	1	DMSO	0.825	1.958	47.24	3.96
100	105.3	2	DMSO	0.825	1.958	47.24	3.96
120	104.5	0.25	DMSO	0.825	1.958	47.24	3.96
120	110	0.5	DMSO	0.825	1.958	47.24	3.96
120	118	1	DMSO	0.825	1.958	47.24	3.96
120	125.4	2	DMSO	0.825	1.958	47.24	3.96
140	120.9	0.25	DMSO	0.825	1.958	47.24	3.96
140	127.2	0.5	DMSO	0.825	1.958	47.24	3.96
140	138.4	1	DMSO	0.825	1.958	47.24	3.96
140	144.4	2	DMSO	0.825	1.958	47.24	3.96
60	58.3	0.25	NMP	0.275	3.105	32.55	4.1
60	62.3	0.5	NMP	0.275	3.105	32.55	4.1
60	63.8	1	NMP	0.275	3.105	32.55	4.1
60	65.8	2	NMP	0.275	3.105	32.55	4.1
80	71.3	0.25	NMP	0.275	3.105	32.55	4.1
80	75.9	0.5	NMP	0.275	3.105	32.55	4.1
80	79.9	1	NMP	0.275	3.105	32.55	4.1
80	83	2	NMP	0.275	3.105	32.55	4.1
100	85.6	0.25	NMP	0.275	3.105	32.55	4.1
100	91.1	0.5	NMP	0.275	3.105	32.55	4.1
100	96	1	NMP	0.275	3.105	32.55	4.1
100	101.6	2	NMP	0.275	3.105	32.55	4.1
120	101.7	0.25	NMP	0.275	3.105	32.55	4.1
120	107.8	0.5	NMP	0.275	3.105	32.55	4.1
120	114.2	1	NMP	0.275	3.105	32.55	4.1
120	121.3	2	NMP	0.275	3.105	32.55	4.1
140	119.1	0.25	NMP	0.275	3.105	32.55	4.1
140	125.9	0.5	NMP	0.275	3.105	32.55	4.1
140	133.2	1	NMP	0.275	3.105	32.55	4.1
140	140.7	2	NMP	0.275	3.105	32.55	4.1
60	63	0.5	DMF	0.161	2.06	38.25	3.82
60	65.7	1	DMF	0.161	2.06	38.25	3.82
60	67.3	2	DMF	0.161	2.06	38.25	3.82
80	75	0.25	DMF	0.161	2.06	38.25	3.82
80	78.2	0.5	DMF	0.161	2.06	38.25	3.82
80	82	1	DMF	0.161	2.06	38.25	3.82
80	85	2	DMF	0.161	2.06	38.25	3.82
100	89.5	0.25	DMF	0.161	2.06	38.25	3.82
100	92.5	0.5	DMF	0.161	2.06	38.25	3.82
100	99.3	1	DMF	0.161	2.06	38.25	3.82
100	103.6	2	DMF	0.161	2.06	38.25	3.82
120	111	0.25	DMF	0.161	2.06	38.25	3.82
120	115.7	0.5	DMF	0.161	2.06	38.25	3.82
120	118	1	DMF	0.161	2.06	38.25	3.82
120	123	2	DMF	0.161	2.06	38.25	3.82
140	129.5	0.25	DMF	0.161	2.06	38.25	3.82
140	133.5	0.5	DMF	0.161	2.06	38.25	3.82
140	140.5	1	DMF	0.161	2.06	38.25	3.82
140	143.6	2	DMF	0.161	2.06	38.25	3.82
60	67	0.25	Water	0.123	4.18	80.1	1.8546

60	67	0.5	Water	0.123	4.18	80.1	1.8546
60	67.7	1	Water	0.123	4.18	80.1	1.8546
60	67.6	2	Water	0.123	4.18	80.1	1.8546
80	82.3	0.25	Water	0.123	4.18	80.1	1.8546
80	84.1	0.5	Water	0.123	4.18	80.1	1.8546
80	86.6	1	Water	0.123	4.18	80.1	1.8546
80	86.9	2	Water	0.123	4.18	80.1	1.8546
100	100.3	0.25	Water	0.123	4.18	80.1	1.8546
100	102.2	0.5	Water	0.123	4.18	80.1	1.8546
100	105.8	1	Water	0.123	4.18	80.1	1.8546
100	107.5	2	Water	0.123	4.18	80.1	1.8546
120	119.2	0.25	Water	0.123	4.18	80.1	1.8546
120	121.7	0.5	Water	0.123	4.18	80.1	1.8546
120	126.2	1	Water	0.123	4.18	80.1	1.8546
120	128.6	2	Water	0.123	4.18	80.1	1.8546
140	136.3	0.25	Water	0.123	4.18	80.1	1.8546
140	142.3	0.5	Water	0.123	4.18	80.1	1.8546
140	147.5	1	Water	0.123	4.18	80.1	1.8546
60	62.6	0.25	Acetonitrile	0.062	2.229	36.64	3.92
60	65.1	0.5	Acetonitrile	0.062	2.229	36.64	3.92
60	65.5	1	Acetonitrile	0.062	2.229	36.64	3.92
60	68.7	2	Acetonitrile	0.062	2.229	36.64	3.92
80	77.8	0.25	Acetonitrile	0.062	2.229	36.64	3.92
80	78.4	0.5	Acetonitrile	0.062	2.229	36.64	3.92
80	82	1	Acetonitrile	0.062	2.229	36.64	3.92
80	87.8	2	Acetonitrile	0.062	2.229	36.64	3.92
100	99.6	0.25	Acetonitrile	0.062	2.229	36.64	3.92
100	92	0.5	Acetonitrile	0.062	2.229	36.64	3.92
100	101.2	1	Acetonitrile	0.062	2.229	36.64	3.92
100	108	2	Acetonitrile	0.062	2.229	36.64	3.92
120	121.5	0.25	Acetonitrile	0.062	2.229	36.64	3.92
120	114.2	0.5	Acetonitrile	0.062	2.229	36.64	3.92
120	120.4	1	Acetonitrile	0.062	2.229	36.64	3.92
120	128.9	2	Acetonitrile	0.062	2.229	36.64	3.92
140	141.3	0.25	Acetonitrile	0.062	2.229	36.64	3.92
140	135.1	0.5	Acetonitrile	0.062	2.229	36.64	3.92
140	142.1	1	Acetonitrile	0.062	2.229	36.64	3.92
140	150.3	2	Acetonitrile	0.062	2.229	36.64	3.92
60	63	0.25	THF	0.047	1.72	7.52	1.75
60	66	0.5	THF	0.047	1.72	7.52	1.75
60	65.1	1	THF	0.047	1.72	7.52	1.75
60	65.8	2	THF	0.047	1.72	7.52	1.75
80	78.7	0.25	THF	0.047	1.72	7.52	1.75
80	82.9	0.5	THF	0.047	1.72	7.52	1.75
80	87	1	THF	0.047	1.72	7.52	1.75
80	84.5	2	THF	0.047	1.72	7.52	1.75
100	93	0.25	THF	0.047	1.72	7.52	1.75
100	99.2	0.5	THF	0.047	1.72	7.52	1.75
100	106.9	1	THF	0.047	1.72	7.52	1.75
100	102.7	2	THF	0.047	1.72	7.52	1.75
120	109.8	0.25	THF	0.047	1.72	7.52	1.75
120	116.2	0.5	THF	0.047	1.72	7.52	1.75
120	127.9	1	THF	0.047	1.72	7.52	1.75

140	130.5	0.25	THF	0.047	1.72	7.52	1.75
140	138.1	0.5	THF	0.047	1.72	7.52	1.75
140	149	1	THF	0.047	1.72	7.52	1.75
60	68.3	0.25	Toluene	0.04	1.707	2.379	0.37
60	71.3	0.5	Toluene	0.04	1.707	2.379	0.37
60	66.4	1	Toluene	0.04	1.707	2.379	0.37
60	60.4	2	Toluene	0.04	1.707	2.379	0.37
80	82.5	0.25	Toluene	0.04	1.707	2.379	0.37
80	90.1	0.5	Toluene	0.04	1.707	2.379	0.37
80	87.5	1	Toluene	0.04	1.707	2.379	0.37
80	81	2	Toluene	0.04	1.707	2.379	0.37
100	93.9	0.25	Toluene	0.04	1.707	2.379	0.37
100	106.4	0.5	Toluene	0.04	1.707	2.379	0.37
100	107.2	1	Toluene	0.04	1.707	2.379	0.37
120	114.4	0.25	Toluene	0.04	1.707	2.379	0.37
120	123.7	0.5	Toluene	0.04	1.707	2.379	0.37
120	127.7	1	Toluene	0.04	1.707	2.379	0.37
140	133.7	0.25	Toluene	0.04	1.707	2.379	0.37
140	142.6	0.5	Toluene	0.04	1.707	2.379	0.37

Scaled models for IR sensor 1 (using scaled variables and y-values).

**Model**	**Adjusted R^2**	**RSE[a]**	**Variable**	**Coefficient**	**p value**
Sensor 1, model 1	0.985	0.125	*set temperature*	0.9753	$< 2\times10^{-16}$ ***
All data			flow rate	$1.208\text{x}10^{-2}$	0.2077
			tan δ	$7.092\text{x}10^{-2}$	6.69×10^{-12} ***
			dielectric constant	$3.317\text{x}10^{-2}$	0.0536
			dipolar moment	$8.578\text{x}10^{-2}$	3.23×10^{-12} ***
			specific heat capacity	$3.324\text{x}10^{-2}$	0.0364
Sensor 1, model 2	0.984	0.126	*set temperature*	0.9743	$< 2\times10^{-16}$ ***
All data			*tan δ**	$7.371\text{x}10^{-2}$	1.30×10^{-12}***
			dipolar moment	$9.863\text{x}10^{-2}$	$< 2\times10^{-16}$ ***
			specific heat capacity	$5.761\text{x}10^{-2}$	1.14×10^{-8}***
Sensor 1, model 3 *Excluded THF and toluene*	0.989	0.108	*set temperature*	0.9942	$< 2\times10^{-16}$ ***
Sensor 1, model 4	0.965	0.186	*set temperature*	0.9839	$< 2\times10^{-16}$ ***
All data			flow	$2.116\text{x}10^{-2}$	0.138
Sensor 1, model 5 *All data*	0.965	0.187	*set temperature*	0.9825	$< 2\times10^{-16}$ ***
Sensor 1, model 6 *High tan δ*	0.986	0.118	*set temperature*	0.9931	$< 2\times10^{-16}$ ***
Sensor 1, model 7 *Medium tan δ*	0.990	0.0978	*set temperature*	0.9953	$< 2\times10^{-16}$ ***
Sensor 1, model 8 *Low tan δ*	0.959	0.202	*set temperature*	0.9797	$< 2\times10^{-16}$ ***
Sensor 1, model 9 *High and medium tan δ*	0.988	0.109	*set temperature*	0.9941	$< 2\times10^{-16}$ ***

[a]Residual standard error. *** Significant at 99.9%level.

Scaled models for IR sensor 2 (using scaled variables and y-values).

Model	Adjusted R^2	RSE[a]	Variable	Coefficient	p value
Sensor 2, model 1 *All data*	0.975	0.16	*set temperature*	0.9872	$< 2\times10^{-16}$ ***
			flow rate	0.1378	$< 2\times10^{-16}$ ***
			tan δ	-8.459×10^{-2}	1.28×10^{-10}***
			dielectric constant	1.043e-01	4.63×10^{-6}***
			dipolar moment	-8.692×10^{-2}	1.38×10^{-8}***
			specific heat capacity	-7.279×10^{-2}	5.17×10^{-4}***
Sensor 2, model 2 *All data*	0.963	0.193	*set temperature*	0.9813	$< 2\times10^{-16}$ ***
			flow	0.1299	1.65×10^{-15}***
Sensor 2, model 3 *All data*	0.946	0.232	*set temperature*	0.9728	$< 2\times10^{-16}$ ***
Sensor 2, model 4 *High tan δ*	0.983	0.129	*set temperature*	0.9780	$< 2\times10^{-16}$ ***
			flow	0.1660	5.42×10^{-14}***
Sensor 2, model 5 *Medium tan δ*	0.959	0.202	*set temperature*	0.9786	$< 2\times10^{-16}$ ***
			flow	0.1478	1.01×10^{-6}***
Sensor 2, model 6 *Low tan δ*	0.970	0.175	*set temperature*	0.9954	$< 2\times10^{-16}$ ***
			flow	9.230×10^{-2}	0.000389***
Sensor 2, model 7 *High and medium tan δ*	0.970	0.173	*set temperature*	0.9777	$< 2\times10^{-16}$ ***
			flow	0.1564	$< 2\times10^{-16}$ ***

[a]Residual standard error. *** Significant at 99.9%level.
